# Culture conditions defining glioblastoma cells behavior: what is the impact for novel discoveries?

**DOI:** 10.18632/oncotarget.20193

**Published:** 2017-08-11

**Authors:** Pítia Flores Ledur, Giovana Ravizzoni Onzi, Hui Zong, Guido Lenz

**Affiliations:** ^1^ Department of Biophysics and Center of Biotechnology, Federal University of Rio Grande do Sul (UFRGS), Porto Alegre, RS–Brazil; ^2^ Department of Microbiology, Immunology, and Cancer Biology, University of Virginia, Charlottesville, VA, USA

**Keywords:** culture conditions, culture media, glioblastoma, heterogeneity, drug discovery

## Abstract

In cancer research, the use of established cell lines has gradually been replaced by primary cell cultures due to their better representation of *in vivo* cancer cell behaviors. However, a major challenge with primary culture involves the finding of growth conditions that minimize alterations in the biological state of the cells. To ensure reproducibility and translational potentials for research findings, culture conditions need to be chosen so that the cell population in culture best mimics tumor cells *in vivo*. Glioblastoma (GBM) is one of the most aggressive and heterogeneous tumor types and the GBM research field would certainly benefit from culture conditions that could maintain the original plethora of phenotype of the cells. Here, we review culture media and supplementation options for GBM cultures, the rationale behind their use, and how much those choices affect drug-screening outcomes. We provide an overview of 120 papers that use primary GBM cultures and discuss the current predominant conditions. We also show important primary research data indicating that “mis-cultured” glioma cells can acquire unnatural drug sensitivity, which would have devastating effects for clinical translations. Finally, we propose the concurrent test of four culture conditions to minimize the loss of cell coverage in culture.

## INTRODUCTION

Glioblastoma (GBM) represents the most common and aggressive primary brain tumor, with a dismal prognosis. Despite standard-of-care treatment, GBM is among the most resistant cancers to radiation and cytotoxic chemotherapy, therefore remaining as an incurable disease with an overall median survival of 15 months [1].

Progresses made in the “omic” areas - genomic, proteomic and so on - have revealed GBM as an extremely heterogeneous disease [2], and many targeted pharmacological agents have been developed since then with the aim to improve current therapies. Unfortunately, the great majority of these drugs have not achieved long-term remissions when tested in animals or even in clinical trials, making treatment options still limited [3].

In the challenge of developing more effective therapeutic strategies, perhaps one of the most important issues to be addressed is to look back on how we are studying this disease and how we are approaching its complexity in the current models of study that we use. In this idea, initial *in vitro* models are crucial, once they serve as a platform for screening novel therapeutic agents, selecting which compounds can and which cannot move forward in the several phases of clinical research, until they finally reach patients. If we do not cover the issues of cellular heterogeneity and of being loyal to the identity of the cells we are studying in *in vitro* assays, substantial information could be misunderstood or even lost in our researches.

### Brief history of tissue culture

#### *In vitro* cell growth establishment

Cell culture is a key technique for cancer research, as it allows scientists to study the biology of tumor cells in an environment with controlled variables. Additionally, due to the ease to scale up and the availability of multi-channel liquid handler, cell culture has become a cost-effective platform for high-throughput drug screening. Rigorous cell culture practice, however, is fundamental for research reproducibility throughout laboratories in the world and for translational potential from bench research into clinical settings.

**Figure 1:**
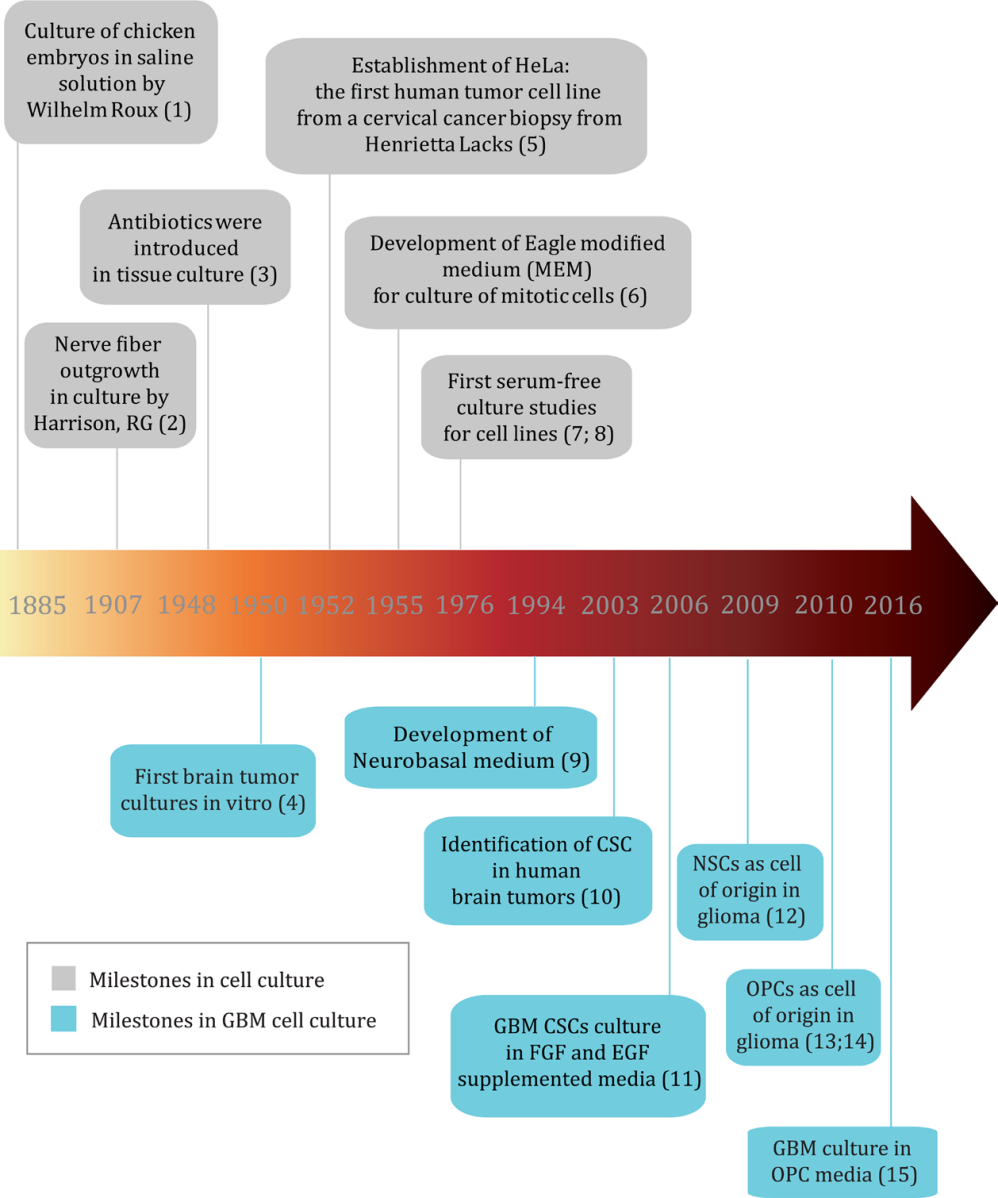
Timeline of important milestones in cell culture and GBM cell culture (reference numbers for milestones can be found in Supplementary File 1)

The history of cell culture can be traced back to late 1800s, when chicken embryos were for the first time maintained alive in a saline solution for several days [4] (Figure 1 - milestones references can be found in Supplementary File 1). Soon after, researchers were able to keep frog and chicken embryo cells alive and growing *in vitro* by using lymph clots [5] and later plasma [6] as nutrient sources. In 1951, a prominent milestone for cell culture was the successful culture of HeLa cells, the first human tumor cell line derived from a cervical cancer biopsy [7]. Using HeLa cells, chemically defined media such as MEM and DMEM were developed and improved, which was a major breakthrough as it avoided the batch-to-batch variation of the animal fluids and thus improved research reproducibility and data comparison among different laboratories [8, 9]. Since then, media supplemented with a source of growth factors has been broadly used to maintain cell lines, and tissue culture has flourished. Among all varieties of growth factor supplements, serum from animal origin, mostly fetal bovine serum (FBS), became the preferred choice because it can sustain most human and animal cell types. Moreover, FBS contains fewer immune system molecules that could interfere with cell growth in culture when compared to serum from a mature bovine immune system [10]. However, FBS components can also vary according to the batch and its components are not fully known, which can lead to low reproducibility and robustness of data generated from cells cultured under this condition [11]. Several serum components present a considerable concentration range among different batches [12], and even growth factors including FGF-2, transforming growth factor β 1 (TGFβ-1) and glial growth factor (GGF) can be added to this list [13]. Such inconsistency could also lead to unwanted effects in culture such as non-specific binding, activation or inactivation of molecules [11, 14], and interfere with biological aspects such as growth capacity and induction of differentiation. Furthermore, ethical concerns with regards to animal rights in the use of serum have arisen [11]. These issues led to the development of serum-free media supplemented with defined growth factors [15].

#### Establishment and evolution of GBM cell culture conditions

Since most cell culture systems have been developed to culture fibroblasts, epithelial and blood cells, the culture of brain cells, including GBM, faces its unique challenges. While fibroblasts, epithelial and blood cells are in contact with serum, brain cells are in contact with cerebrospinal fluid (CSF), that has a distinct protein composition, as many serum proteins are unable to cross the blood-brain barrier [16]. Although the majority of CSF proteins originate from the blood, about 20% of them are brain derived [17]. Also, basal media such as DMEM and DMEM/F12 were developed in order to promote rapid cell division of somatic cells, and therefore were not developed for post-mitotic cells such as neurons.

Neurobasal media was the first media developed for neurons [18], based on DMEM with reduced osmolarity and lower glutamine concentrations. Excitatory amino acids were also eliminated to avoid neurotoxicity. Moreover, serum-free supplements for neuronal and glial cell culture started to be developed, such as B27 and N2. These supplements contain basic molecules needed for neuronal growth such as vitamins like biotin (B27) and proteins like insulin and transferrin (B27 and N2). Some of them are also composed of growth factors, including EGF and FGF, as is the case of G-5 supplement (ThermoFischer^®^), developed for the culture of glial cells of astrocytic phenotype (normal and tumor) [15]. B27 and the combination of Neurobasal media with B27, for instance, allowed long-term survival with high cell viability for hippocampal neurons [18].

While the primary consideration of media/growth factor/hormone choices is to sustain the proliferation and viability of cultured cells, it should be noted that some components can significantly skew cellular behaviors from their *in vivo* biology and this has been shown to be true for neural cells. A good example is that of mature astrocytes. After being exposed to serum, astrocytes have long lasting gene expression changes that remain even after serum withdrawal [16]. To improve this scenario, Foo and collaborators have shown that mature astrocytes can be successfully cultured in serum-free media in the presence of HBEGF and vascular cells, maintaining their gene expression profile much closer to that of astrocytes *in vivo*. Another example regarding neurophysiological activity when culturing mature neurons *in vitro*, where even small adjustments in media composition - such as inorganic salts, energetic substrates and amino acid concentration - can lead to better action potential and synaptic communication [19].

The first brain tumors were cultured *in vitro* in the 50s [20], and their culture prospered in the beginning of the 60s [21]. In 1968, Pontén and Macixtyre initiated the Uppsala (U) series of malignant glioma (MG) cell lines by establishing four cell lines derived from human malignant gliomas, still widely used by researchers in the glioma field [22]. Researchers from the Uppsala University have found also that one of the most used GBM cell lines, U87MG, obtained from American Type Culture Collection (ATCC) was quite different from that collected from the original tumor. Despite sharing transcriptional features of brain tumors, ATCC's U87MG has an unknown source. This incident highlighted the urgent need for researchers to carefully validate the cell lines used in their works [23].

Glioma had been traditionally cultured in FBS-enriched DMEM media, the prevalent method in the past. However, in 2003, the description of cancer stem cells (CSCs) in GBM [24] raised serious concerns about serum use since it can induce neural stem cell (NSC) differentiation [25], and CSCs were thought to arise from NSCs [26]. Ever since, the search for a medium to better preserve the phenotype of patient-derived glioma cells began [24, 26]. In 2006, inspired by NSC culture conditions, the Fine lab used serum-free, EGF/FGF-2-supplemented Neurobasal medium to cultivate primary glioma cells and found that these cells remained more similar to the parental tumors than those cultured in serum-containing DMEM medium [27]. They observed that serum-cultured GBM cells had limited growth, responded less to differentiation stimulus and presented genomic alterations not found in the original tumors. In contrast, GBM cells maintained in Neurobasal medium supplemented with EGF/FGF-2 retained the same proliferation capacity, migration/invasion histological features, genotype and gene expression profile of the tumors from which they were obtained. More recently, the Uhrbom lab has established a biobank of glioma cell lines (The Human Glioblastoma Cell Culture Resource) with over sixty cell lines from surgical GBM samples, by growing them first as spheres and then as monolayer cultures in EGF/FGF-2 enriched media [28, 29]. Most of the cell lines produced in this manner were classified at the same proportions as the parental tumors, with a few exceptions [29]. These findings demonstrate the importance of tailoring culture conditions based on the biological properties of cell types in culture. In the next sections, we review the most common culture media used for glioma and how to customize media options for a particular biological question.

### Review of the growth conditions used in GBM primary cultures

**Figure 2:**
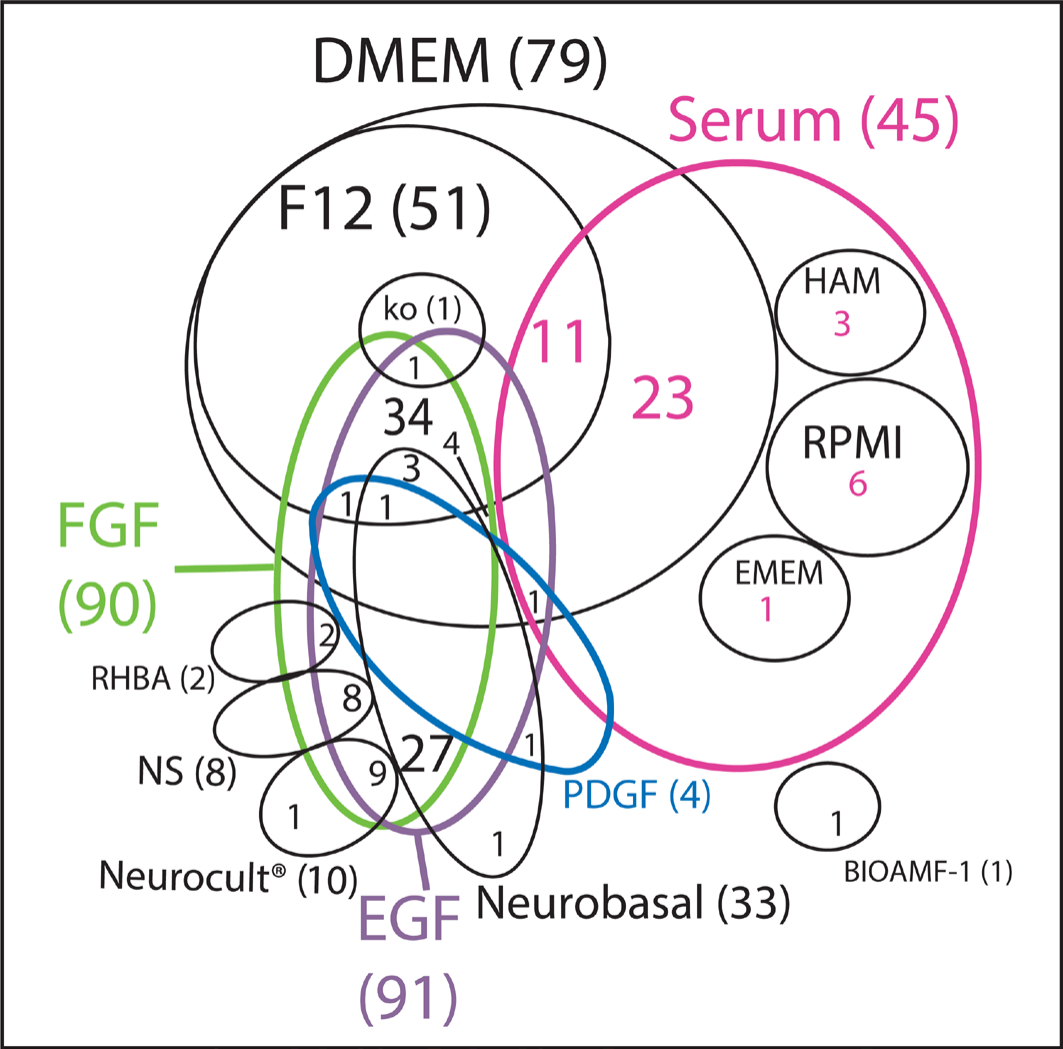
Literature review of growth conditions used in GBM primary cultures Venn diagram of the most used culture media (in black) and the respective supplementation factors (different colors) used in GBM primary cultures. NS: non-specified basal medium. Ko: DMEM/F12 knockout.

We reviewed the literature to gather information on the most commonly used conditions for culturing GBM primary cells. Data collected from 120 papers show that DMEM as base medium, alone or mixed with Ham's F12 nutrient mixture, and Neurobasal medium are the most common choices of medium to culture these cells in (Figure 2 and Supplementary File 2). DMEM and F-12 are standard types of media, broadly used in mammalian cell culture. Neurobasal media, as already discussed in this review, was designed to meet the neuronal cell requirements without the need of an astrocyte feeder layer [18]. These media are basically composed of glucose, amino acids, vitamins and inorganic salts, in specific and controlled concentrations. For the full list of papers reviewed to investigate the most common primary culture conditions, please refer to Supplementary File 2.

Although medium choice has an impact in cell behavior, growth factor supplementation is also fundamental to maintain cell metabolism and promote specialized cell functions. Our search shows that serum is still widely used in GBM research, representing close to half of the studies when compared to EGF/FGF-2 (Figure 2). This may be because serum as a rich nutrient source could potentially embrace a higher variety of cell types than media containing one or two isolated growth factors. Notwithstanding, certain types of glioma have proven hard to maintain *in vitro* even when cultured in the presence of serum, such as the IDH1 mutant gliomas [30]. Therefore, it is not surprising that growing biopsies in NSC media, where a narrower variety of growth factors is provided, has been such a challenging task. Although NSC media seems to be a better choice than serum in terms of maintaining some of the parental tumor properties [27], at the same time it does not work for all glioma samples. Gunther and collaborators were able to culture only 9 out of 19 glioma biopsies for longer than 8 passages in NSC media [31]. Galli and collaborators found a similar proportion, 6 out of 12 tumors were well-established *in vitro* [32] under the same conditions. This indicates that NSC conditions are not always ideal for keeping cells alive in culture, especially when we consider different cells of origin, as discussed below. Our observations show that there is a higher initial growth efficacy of primary GBM tumors in serum-containing media when compared to defined medium, although serum-containing media seems to induce a higher rate of senescence.

The complexity of the Venn diagram shown in Figure 2 indicates that, although some culture conditions are clearly preferred, there are over 20 different culture conditions published; therefore, there is far from consensus on the ideal media to grow primary tumors. The vast array of conditions employed makes the comparison of studies much more difficult, adding to the already challenging heterogeneity of gliomas. Unfortunately, none of the papers reviewed explained the rationale behind the choice of growth factor combination.

Thirteen papers compared differentiating and non-differentiating growth conditions concomitantly in GBM samples. Among the 28 different biological features evaluated, 4 were not significantly different, 10 were higher in the differentiated cells and 14 were higher in the non-differentiated cells (Supplementary File 3). Among the differences found, GBM cells in non-differentiating culture conditions displayed higher invasive potential [33], lower drug efflux capacity [34] and higher sensitivity to immune responses mediated by NK and T-cells [35] when compared to their more differentiated counterparts.

The differentiating culture condition most frequently used was media supplemented with 10% FBS while the most common non-differentiating condition was serum-free media, usually supplemented with FGF-2 and EGF. However, even the concentrations of each growth factor varied among the “two-conditions” papers, as well as among all the other papers that used these factors. Standardizing culture conditions would be critical to improve the comparative analysis among all published studies.

### Maintaining the original conditions of cells in culture

The habitat of a cancer cell in a solid tumor is a complex niche that includes immune cells, blood vessels, and dynamic pH and oxygen levels, besides the heterogeneity of the tumor population *per se* [36, 37]. This ecosystem still cannot be reproduced *in vitro*, but some variables such as choice of substrate where cells adhere to, nutrients, growth factors and levels of oxygen can be optimized.

GBM tumor cells in 3D cultures can behave in a different way from cells in a 2D environment [38, 39], favoring the idea that 3D conditions can better mimic what happens in an *in vivo* situation. However, the Kornblum group recently established a gliomasphere bank from 68 patient-derived GBM samples [40] and found a limited correlation between gliomaspheres and their parent tumors as it relates to patterns of gene expression of molecular GBM subtypes, although the model allowed the identification of novel genes of malignancy. Moreover, we have shown that the OPC-glioma model, even though cultured in a 2D manner, shows features considered as “stem-like”, such as formation of spheres, self-renewal, differentiation capacity and the ability to form tumors that recapitulate the parental tumor [41, 42]. Notwithstanding, the discussion of whether to grow GBM in suspension as spheres (3D), or as an adherent culture (2D) is too broad to be addressed in the present review, but it certainly is fundamental to be considered in the design of the culture condition.

When culturing tumors of a high cellular heterogeneity such as GBMs, it is reasonable to consider that the more media/growth factors combinations used, the more likely we are to cover a wider range of this heterogeneity. Nevertheless, when there is a target cell population it is also possible to tailor culture conditions to fulfill the requirements of that specific population. This is the case, for example, of using defined media that favors a non-differentiated state - *i.e*. when working with cancer stem cells and cells of origin - and also of using serum-containing media for more differentiated cells [27, 43]. Despite the well-documented pro-differentiation properties of serum [27, 43], its broader range of growth factors and hormones could favor cell survival and even increase heterogeneity when compared to media with defined growth factors, which may justify the close to 50% of papers published using this condition.

#### Cancer stem cells (CSCs)

In most cancer types, cells with markers of stemness are generally identified as having a higher proportion of grafting rate and resistance capacity than their more differentiated counterparts. However, markers used to identify these cells and the stability of their expression has brought controversy to the field [44, 45].

The similarity of CSCs to normal NSCs led to the hypothesis that human glioma may originate from this cell type. Moreover, normal NSCs express EGFR and therefore, it seemed only natural to keep glioma cells in NSC media *in vitro* [24, 32], which has EGF and FGF-2 as the main growth factors [46, 47]. NSC media became the serum-free standard media for primary cultures in GBM research since then (see Figure 2), and several drug assays have been performed in this media, as a way to search for specific ways of steering CSCs towards death or differentiation [48–51]. Nevertheless, CSC targeting has proven much more challenging than anticipated, since this subpopulation has also been described as extremely heterogeneous [2, 52] and as having a dynamic phenotype [53–55], making its differentiation or elimination a very difficult task. Moreover, there are still inconsistencies in the literature regarding the enrichment of CSCs in NSC media [56], although this could be caused by technical issues, such as the markers used to isolate and/or to analyze the cells or the media used to culture them in.

#### Cell of origin

**Figure 3:**
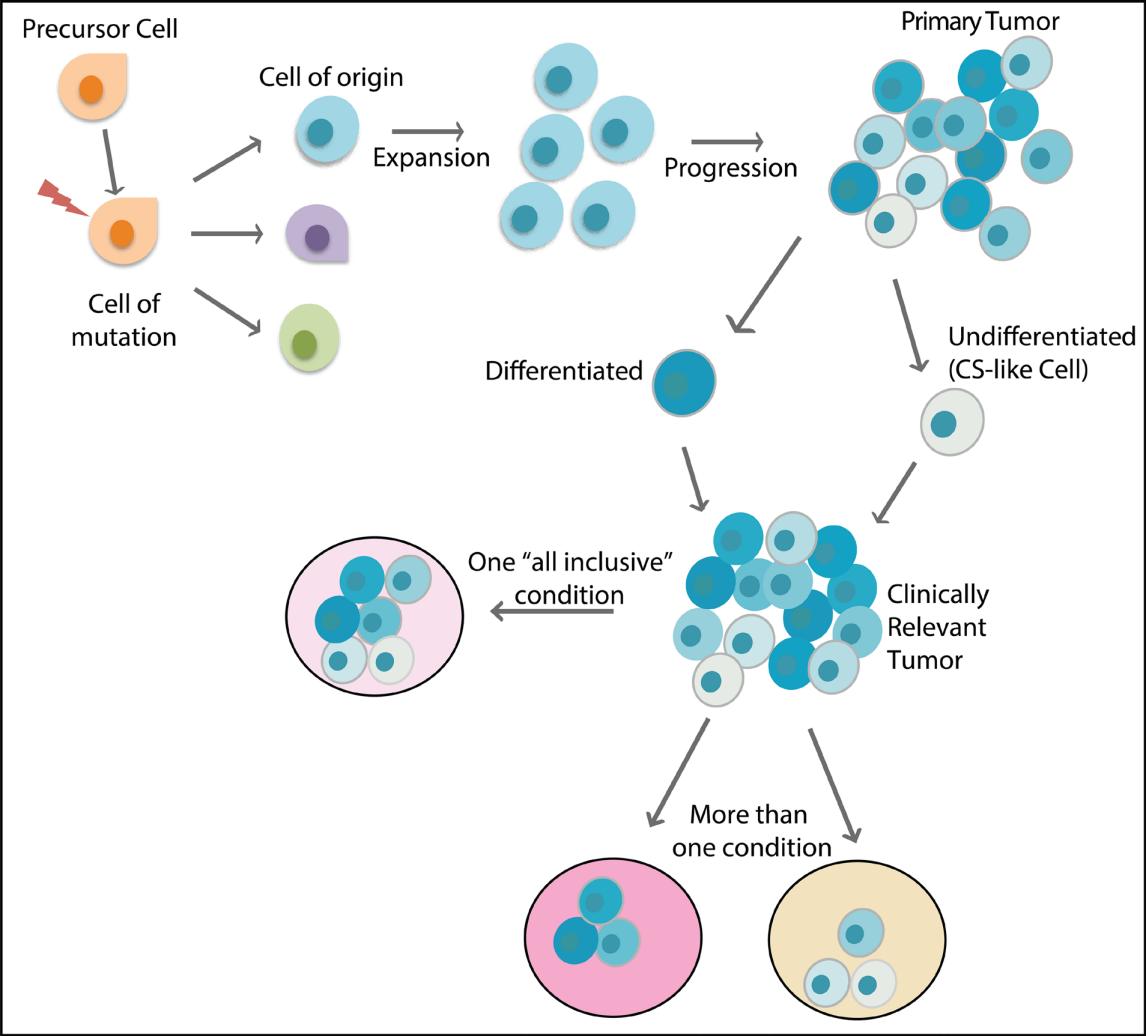
The main players in the evolution of a cancer The cell that undergoes the first mutation event is considered the cell of mutation, which may or may not undergo transformation to originate a tumor. The cell of origin is the specific cell type that is capable of undergoing transformation that generates the tumor mass. The tumor mass is very heterogeneous, with more differentiated (darker blue colors) and less differentiated cell (lighter blue colors), and it is not clear which cells are responsible for establishing metastasis or new tumors in animal models. To maintain the heterogeneity of clinically relevant tumors, either an “all-inclusive” condition or more than one condition must be used separately.

The cell of origin is the cell that undergoes transformations that lead to cancer. The concept of cell of origin emerged from the observation that a certain type of cancer can have different subtypes with distinct cellular and molecular characteristics, and therefore could probably result from different cells of origin [57]. Genetic animal models have been crucial for the investigation of the origin, as specific promoters can drive genetic alterations only in certain target cells. Glioma, for instance, have been shown to have several origins, from progenitor cells [58] to more specialized cells in the brain, such as astrocytes and even neurons [59–61]. Genetic studies have shown that the introduction of mutations in NF1 and p53 in both embryonic and adult NSCs can lead to gliomagenesis [62, 63], as well as the inactivation of p53 and PTEN in the same cell type [64]. In other models, oligodendrocyte precursor cells (OPCs) - a glial cell progenitor - act as cell of origin [42, 65, 66], giving rise to tumors that resemble human oligodendroglioma or the proneural subtype of GBM [67, 68]. A recent conceptual advancement in the field is that the cell that initiates the tumor may be a different cell from the one that undergoes the first mutation event [42, 57, 69] because the latter may not have the signaling context for malignant transformation. It is important to note that the cell of origin concept is different from the CSC concept. CSCs refer to a rare population of tumor cells within the tumor mass that serves as the root (highest hierarchy), which gives rise to less malignant cells to propagate the tumor mass. On the other hand, the cell of origin refers to a normal cell type, which could initiate the tumor when oncogenic mutations occur [57, 70, 71] (Figure 3). The differences between cells of origin in cancer and CSCs as well as some of the best methodologies to characterize and distinguish them were recently reviewed [57, 72].

It has been shown that growing glioma cells based on the knowledge about the cell of origin can maintain some of the original properties of the cells, such as morphology and expression of certain markers. If we consider tumors that have OPCs as their cells of origin, we should use OPC media supplemented with platelet-derived growth factor (PDGF), which has been shown to sustain OPC proliferation and development *in vitro* [41, 42, 73, 74].

Previously, we tested whether OPC-originated glioma cells could preserve their elemental characteristics in culture conditions tailored for OPCs *in vitro*. To test this idea, OPC-originated mouse glioma cells were cultured in conditions for normal OPCs or NSCs for multiple passages. We found that OPC-media cultured glioma cells maintained tumorigenicity, gene expression profiles, and morphologies similar to freshly isolated tumor cells. In contrast, NSC-media cultured glioma cells gradually lost their OPC features and most tumor-initiating ability, and acquired heightened sensitivity to temozolomide [41]. Recently, Jiang et al. have separated patient-derived GBM cells based on a cell of origin signature, and have shown that cells from different origin present distinct sensitivity to drugs [28].

In our literature search, we also found two papers that cultured primary glioma cells in media supplemented with a mixture of EGF, FGF-2 and PDGF [75, 76]. Perhaps this type of media supplementation can be suitable for keeping alive a broader range of less differentiated glioma cells when compared to serum-enriched media, and should therefore be considered according to the research goal.

Therefore, to improve translational potential of glioma research, it is important to identify the cell-of-origin and subsequently consider this knowledge to establish culture conditions that allow the preservation of native properties of tumor cells.

### The role of *in vitro* culture for drug screening

Given the importance of *in vitro* tests for initial screenings of new drugs, cell culture models have increased in complexity with the aim to better mimic what happens to tumors *in vivo*, trying to reduce experimental uncertainties and anticipate possible effects of these new compounds. Novel *in vitro* models have been developed, such as 3D cell culture, tissue engineering, biomaterials, microfluidics, allowing us to incorporate different cell types and extracellular matrix components to cultures, as well as to control spatial and temporal introduction of soluble factors [77].

**Figure 4:**
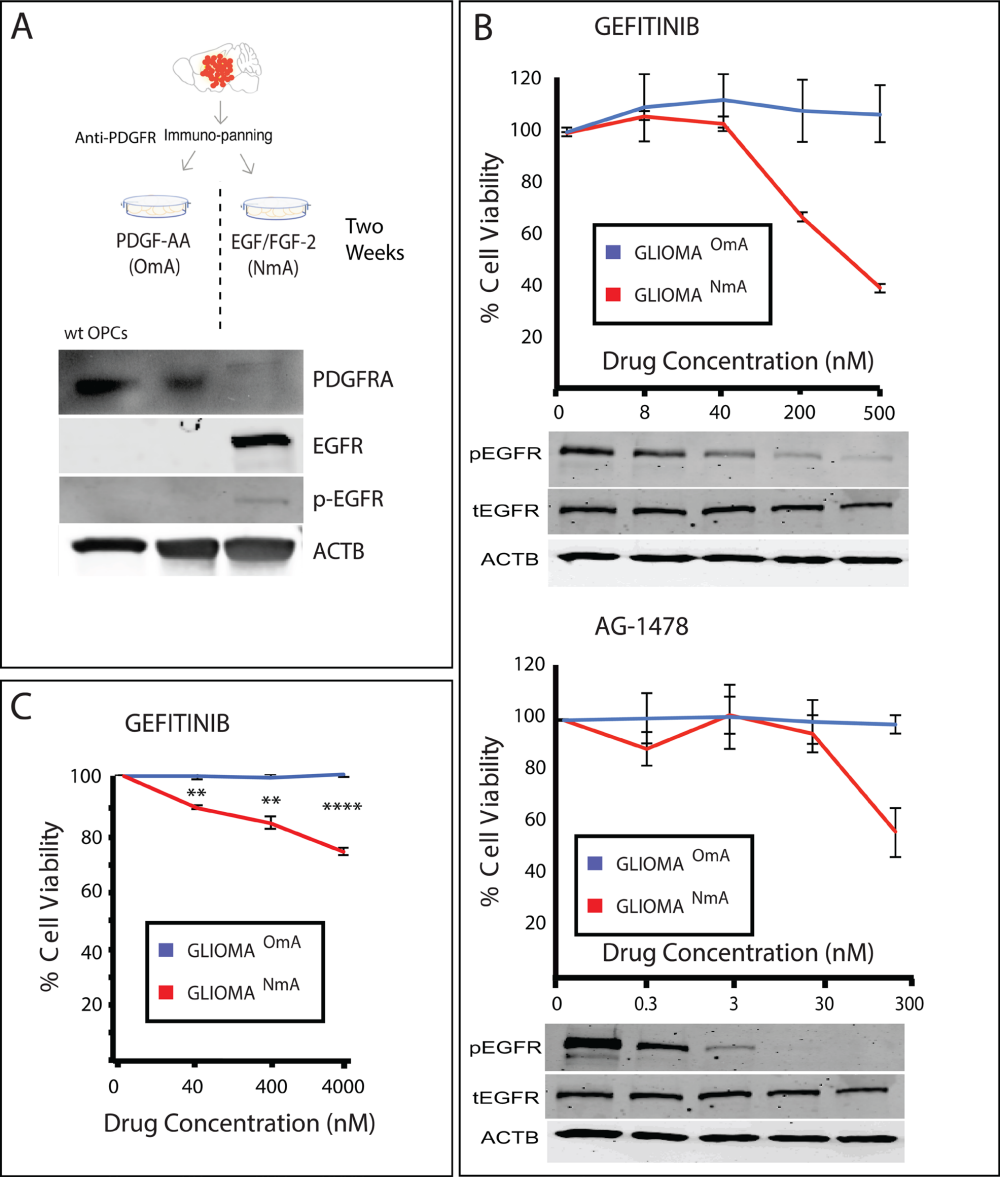
Culture conditions can interfere with response to drugs (**A**) Mouse glioma was dissociated, purified through immunopanning and cultured in OPC media (containing PDGF as growth factor) and NSC media (containing EGF/FGF-2 as growth factors). Wild type (wt) OPCs were purified through immunopanning and used as control. PDGFR expression, naturally present in wt OPCs, is reduced when cells are cultured in NSC media, and EGFR starts to be expressed in an active (phosphorylated) form. (**B**) EGFR inhibitors Gefitinib and AG-1478 reduce cell viability of treated cells only when they are cultured in NSC media (NmA– NSC media accustomed cells), and not in OPC media accustomed cells (OmA). pEGFR = phosphorylated EGFR, tEGFR = total EGFR. Values were normalized based on a non-treated control (vehicle–DMSO-only). (**C**) A similar response is seen in a human GBM primary cell line accustomed to both NSC media and OPC media and treated with Gefitinib. Samples were normalized based on non-treated controls. **p ≤ 0.01; ****p ≤ 0.0001.

However, besides making use of these high complexity models, simpler cell culture concepts and ideas can be further explored to avoid misinterpretation of results, which is crucial when screening new drugs. One question to debate is about the culture medium used in the tests. Since all drug screenings depend upon an *in vitro* step prior to moving to *in vivo* assays and then clinical trials [78], the media in which cells are maintained might have a pivotal role in the choice of which drugs go forward in the pre-clinical setting towards clinical development. We have recently shown that cells from a GBM model driven by a promoter that is active in OPCs were more resistant to TMZ when cultured in media suitable for this cell type than the same cells cultured in NSC media [41]. Additionally, growing these cells in NSC media containing EGF and FGF-2 increased the expression and signaling of EGFR and the acquired sensitivity to selective EGFR inhibitors (Figure 4). It is important to mention that this model resembles the proneural subtype of GBM and that tumors with other molecular profiles may present different responses.

Although a similar trend was observed in a human GBM line (Figure 4C), human GBMs are more heterogeneous and complex than a genetic model and could present subpopulations of cells that express different receptor tyrosine kinases (RTKs), or even more than one RTK simultaneously [79], which could lead to different possible scenarios. It is also important to stress that there is no easy test to determine which condition produces responses more similar to the response of the tumor but the fact that different growth conditions lead to different pharmacological results suggests that cells with these conditions or the plasticity to adopt these conditions are present and could potentially be expressed in the tumor [79]. This example should serve as a cautionary note for cell-based drug screening efforts as mis-cultured cells could lead to drug hits that might be ineffective to the original tumor cells and that would go forward in the drug development process.

### Final remarks

**Figure 5:**
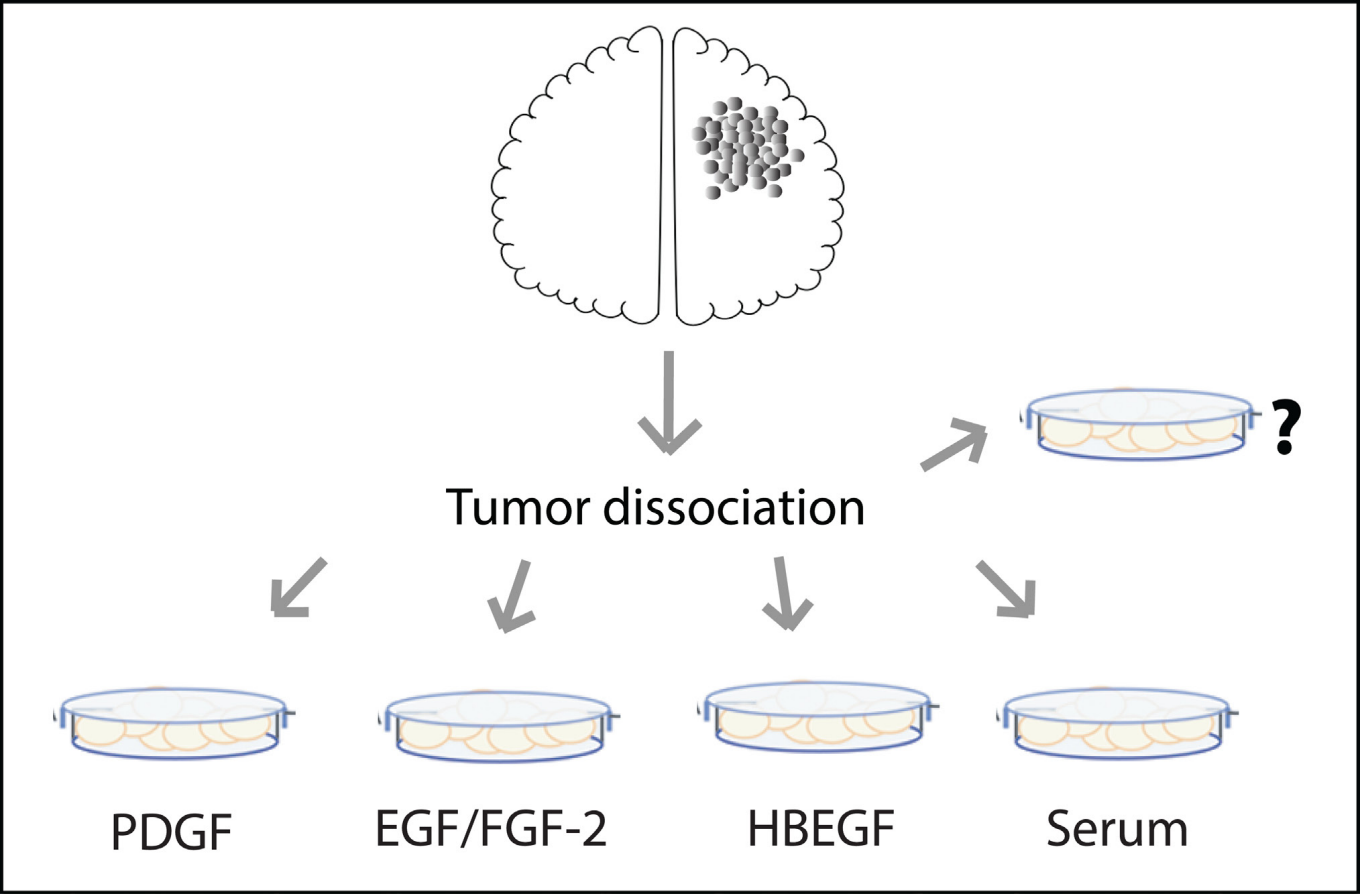
Schematic representation of media choice options available for GBM primary culture To select all cell types possible, one can grow GBM cells in NSC media (with EGF and FGF-2 as growth factors), in PDGF-enriched media (a.k.a. OPC media), in astrocyte specific media (HBEGF as the main growth factor) or in Serum-enriched media. Other possibilities might also be considered.

Whether GBM drug screenings are performed in serum or NSC media, the most commonly used media for *in vitro* GBM culture and research (Figure 2), there is room for improvement, as GBM heterogeneity is very high [2] and culture conditions are selective [41]. The most up to date classification of glioma in Proneural, Neural, Classical and Mesenchymal subtypes based on molecular signatures [67, 80] might not be representative of individual tumors, but only of a majority of cells within the region of the analyzed biopsy [2]. Tumors are genotypically unstable [81–84], and different subtypes could imply different cells of origin. These scenarios raise an important question in terms of how to maintain gliomas *in vitro*. Usually, biopsies are dissociated and cultured in one media choice only, which can work for a specific hypothesis. However, when taking drug discovery into account, as previously discussed (see Figure 4 and references 28, 41, 85), an approach to be considered is to culture cells in all media choices available for glioma (i.e., NSC media, OPC media and FBS containing media). Other media possibilities might also be considered, such as the one adapted for mature astrocytes where HBEGF is used as growth factor [16], as suggested in Figure 5 and; then perform drug screenings in each media option. The drugs that perform best on all choices simultaneously should be the ones moving forward to clinical research. This approach could also be applied to other tumor types, and perhaps drug discovery studies could greatly benefit from this strategy, thus improving the process of developing more efficient drugs to patients.

## MATERIALS AND METHODS

### Search details

We performed an electronic search (last updated on May 2nd, 2017) for papers indexed in the Web of Science database. The search strategy comprised the terms “GBM primary culture” and papers published between 2012–2017. For inclusion in this review, papers had to describe the conditions in which GBM primary cells were cultivated *in vitro* regarding media choice and/or growth factor supplementation. No language restriction was applied. By this search strategy, 175 papers were identified. After reviewing their abstracts and their full-text form, 120 eligible papers were chosen and examined. Exclusion criteria:

- papers which did not used GBM cells;
- papers which used only commercial GBM cell lines instead of primary cultures;
- papers which did not describe the conditions used to grow primary GBM cells;

### Cell culture

Mouse cells used in Figure 4 were purified from tumors from MADM (TG11, GT11), hGFAP-Cre, p53KO, NF1 flox or MADM (TG11, GT11), NG2-Cre, p53KO, NF1 null mice. Cells were purified through an immunopanning procedure [42] and cultured in either FGF-2 and EGF enriched media or in PDGF-AA enriched media as previously described [41]. Animal procedures followed animal care guidelines, under University of Virginia IACUC, approval #3955. Human glioma use was approved by the University of Virginia Hospital institutional review board under protocol IRB-HSR#17626. After biopsy collection, cells were dissociated and cultured in the same media as the mouse cells and as previously described [41].

### Western blotting

Cells were lysed in lysis buffer 17 (R&D, 895943), supplemented with protease inhibitor cocktail tablets (Roche, 11836153001) and Halt phosphatase inhibitor (Thermo Scientific, 1862495). Total protein was adjusted according to concentration measured by Pierce BCA protein assay kit (Thermo Scientific, cat. # 23227). Protein samples were subjected to SDS-PAGE and transferred to polyvinylidenedifluoride membranes.

### MTT assay and drug treatments

Glioma^NmA^ and Glioma^OmA^ cells were plated at a density of 2 × 10^5^ cells/well in 96-well plates and treated with various concentrations of EGFR inhibitors Gefitinib and AG-1478 (Selleckchem), as indicated. Control samples were treated with the vehicle used to dilute drugs with (DMSO), and treated samples were normalized based on non-treated control. MTT assay was used to assess cell viability 48 hours after treatments.

## SUPPLEMENTARY MATERIALS









## Abbreviations

GBM: Glioblastoma
CSC: Cancer stem-like cell
NSC: Neural stem cell
OPC: oligodendrocyte precursor cell
EGF: epidermal growth factor
EGFR: epithelial growth factor receptor
HBEGF: Heparin-binding EGF-like growth factor
FGF: fibroblast growth factor
PDGF: Platelet -derived growth factor
TGFβ-1: transforming growth factor β 1
GGF: glial growth factor
CSF: cerebrospinal fluid
FBS: fetal bovine serum
IDH1: isocitrate dehydrogenase
EFEMP2: EGF Containing Fibulin Like Extracellular Matrix Protein 2
LGALS8: Lectin, Galactoside-Binding, Soluble,8
NmA: NSC media accustomed cells
OmA: OPC media accustomed cells
