# Supplementary material for: Culture conditions defining glioblastoma cells behavior: what is the impact for novel discoveries?
Source: Oncotarget. 2017 Aug 11;8(40):69185–97. doi: 10.18632/oncotarget.20193 (PMC5620329; doi:10.18632/oncotarget.20193)
Supplement: Supplementary file 1 [file oncotarget-08-69185-s001.pdf]

Culture conditions defining glioblastoma cells behavior: what is the impact for novel discoveries?

SUPPLEMENTARY MATERIALS

Supplementary File 1: List of references cited in milestones addressed in Figure 1 -Timeline of important milestones in cell culture and GBM cell culture. See Supplementary_File_1

Supplementary File 2: Literature review of growth conditions used in GBM primary cultures. See Supplementary_File_2

Supplementary File 3: Qualitative summary of the data published in papers comparing the effect on several characteristics of differentiating versus non-differentiating culture conditions. See Supplementary_File_3
