# Supplementary material for: Culture conditions defining glioblastoma cells behavior: what is the impact for novel discoveries?
Source: Oncotarget. 2017 Aug 11;8(40):69185–97. doi: 10.18632/oncotarget.20193 (PMC5620329; doi:10.18632/oncotarget.20193)
Supplement: Supplementary file 2 [file oncotarget-08-69185-s002.docx]

References for Figure 1:

1. Langdon SP. Cancer Cell Culture. New Jersey: Springer Science & Business Media, 2004.

2. Harrison RG, Greenman MJ, Mall FP et al. Observations of the living developing nerve fiber. The Anatomical Record 1907;1:116–28.

3. Keilová H. The effect of streptomycin on tissue cultures. Cellular and Molecular Life Sciences, 1948.

4. Kersting G. Tissue Culture of Human Gliomas. Karger Publishers, 1968:165–202.

5. Scherer WF, Syverton JT, Gey GO. Studies on the propagation in vitro of poliomyelitis viruses. IV. Viral multiplication in a stable strain of human malignant epithelial cells (strain HeLa) derived from an epidermoid carcinoma of the cervix. J Exp Med 1953;97:695–710.

6. Eagle H. Nutrition needs of mammalian cells in tissue culture. Science 1955;122:501–14.

7. Ham RG. Clonal growth of mammalian cells in a chemically defined, synthetic medium. Proc Natl Acad Sci U S A 1965;53:288–93.

8. Hayashi I, Sato GH. Replacement of serum by hormones permits growth of cells in a defined medium. Nature 1976;259:132–4.

9. Brewer GJ, Torricelli J, Evege EK et al. Neurobasal medium/B27 supplement: A new serum-free medium combination for survival of neurons. Focus 1996:1–4.

10. Singh SK, Clarke ID, Terasaki M et al. Identification of a cancer stem cell in human brain tumors. Cancer Res 2003;63:5821–8.

11. Lee J, Kotliarova S, Kotliarov Y et al. Tumor stem cells derived from glioblastomas cultured in bFGF and EGF more closely mirror the phenotype and genotype of primary tumors than do serum-cultured cell lines. Cancer Cell 2006;9:391–403.

12. Wang Y, Yang J, Zheng H et al. Expression of mutant p53 proteins implicates a lineage relationship between neural stem cells and malignant astrocyticglioma in a murine model. Cancer Cell 2009;15:514– 26.

13. Persson AI, Petritsch C, Swartling FJ et al. Non-stem cell origin for oligodendroglioma. Cancer Cell 2010;18:669–82.

14. Liu C, Sage JC, Miller MR et al. Mosaic analysis with double markers reveals tumor cell of origin in glioma. Cell 2011;146:209–21.

15. Ledur PF, Liu C, He H et al. Culture conditions tailored to the cell of origin are critical for maintaining native properties and tumorigenicity of glioma cells. Neuro Oncol 2016;18:1413–24.
